# Testing the Single and Combined Effect of Kaolin and Spinosad against *Bactrocera oleae* and Its Natural Antagonist Insects in an Organic Olive Grove

**DOI:** 10.3390/life13030607

**Published:** 2023-02-22

**Authors:** Veronica Vizzarri, Luca Lombardo, Carmine Novellis, Pierluigi Rizzo, Massimiliano Pellegrino, Giuseppe Cruceli, Gianluca Godino, Francesco Zaffina, Annamaria Ienco

**Affiliations:** CREA Research Centre for Olive, Citrus and Tree Fruit, 87036 Rende, Italy

**Keywords:** *Bactrocera oleae*, spinosad, kaolin, organic oliviculture

## Abstract

The presence and infestation level of *Bactrocera oleae* was monitored in an organic olive orchard divided into differently treated parcels with kaolin (K), spinosad (S) and with kaolin and spinosad (K + S) in alternate rows. The treatments did not seem to affect olive fruit fly population dynamics, while statistically significant protective effects were recorded against total and harmful infestation, but not against the active one. Eventually, neither kaolin nor spinosad were shown to have a particular detrimental effect against naturally occurring *B. oleae* parasitoids.

## 1. Introduction

Olive fly (*Bactrocera oleae,* Rossi) is the most important pest of olive in the Mediterranean Basin [1]. The direct damage is caused by larval feeding on fruits, determining quantitative (premature fruit drop and pulp destruction by grub) and qualitative (increase in acidity and peroxide levels and decrease of polar phenols) losses, resulting in relevant production shrinkages reaching up to 80% for oil and 100% for table olives in particularly susceptible cultivars [1,2,3,4]. It is a polyvoltine monophagous species, feeding exclusively on fruits of the genus Olea, whereas its main host plant is *O. europaea* subsp. *europaea* (cultivated and var. *sylvestris*). As such, the evolution and distribution of *B. oleae* is closely linked to the millennial history of this species following its domestication and diffusion [5]. Accordingly, Theophrastus (IV–III century BC) described (*Historia plantarum*, IV, XIV) a worm (σκώληξ) that insinuates into the pulp up to the stone of the olive drupes. The same observation (*vermiculationem*) was reported in the I century AD by Pliny the Elder (*Naturalis Historia*, XVII, XXXVII, 230). In spite of this, the first written reference to the responsibility of a fly for these worms generated in olives is due to Sieuve in 1769 [6], inasmuch as he believed that oviposition occurred on the trunk with the larvae subsequently reaching the drupes. Only a few years later (1773), Grimaldi [7] reported the observations of Calabrian olive growers according to whom the fly laid its eggs directly in the drupe. The correct identification of the pest led to the development of the first forms of control, including anticipated harvest and the employment of mixtures based on cobalt, potassium arsenite or tar [8]. With the advent of synthetic insecticides, the management of olive fly was mainly based on the use of organophosphates (OPs) in cover and bait sprays (e.g., dimethoate and fenthion), while more recently there has been an increase in the use of pyrethroids [1]. Nevertheless, the development of mechanisms of resistance, as well as the evidence of negative effects on the agricultural useful entomofauna and on human health due to the presence of residues in the oil, raised several concerns, resulting in the ban of several OPs worldwide [9,10]. Paradigmatic in this sense for olive growing is the case of the cytotropic insecticide dimethoate, which was abundantly and predominantly used for decades against *B. oleae,* and whose employment was revoked in the EU in 2020 due to failure to renew the European approval pursuant to regulation (EU) 2019/1090, as it was not possible to exclude its genotoxic potential (as well as the one of its main metabolite, methoate which is an *in vivo* mutagenic agent). The Authority also concluded that there is a dimethoate-correlated high risk for mammals and non-target arthropods (including honey bees). The selection of alternative control methods with little or no environmental impact is therefore becoming a pressing challenge for oliviculture. The pursuit of effective systems to contrast the olive fly is particularly important in the organic sector. In Italy, the organic surface planted with olive trees is equivalent to 242,708 ha, approximately 22% of the total, divided by over 37,000 farms (certified or in conversion phase [11]). However, the production of organic oil in the Italian market covers only slightly less than 10% of the total, corresponding to approximately 28,000 Mg. This gap can be attributed not only to the reduced presence of oil mills equipped for biological production, but also to the general lower production of organic surfaces (actually due to the reduced alternatives on the market that are allowed to be used for olive pathogen control) [12,13], and to the fact that only the commercial categories of virgin (VOO) and extra virgin (EVOO) olive oils can be labeled as organic [14], so that if *B. oleae* is not effectively countered, the risk of organoleptic defects and chemical changes in oil composition is high. In this regard, natural-origin and biological insecticides are now acquiring more and more interest because of their generally more environmental-friendly action and of their allowed use in organic farming (an exception is represented by rotenone, widely used also against olive fly, that in spite of its natural origin, has been excluded from the list of products allowed in organic farming in Europe, according to Annex I of EC Directive 91/414/2008 because of its high toxicity towards fish and bees). Among the naturalytes, spinosad, an environmentally safe molecule with an attractive and insecticidal action, obtained from the bacterium *Saccharopolyspora spinosa* (Mertz &Yao) through fermentation, has proven its effectiveness in dozens of crops to control several insect pests, including *B. oleae* [1,15,16,17,18,19]. It is a mixture of spinosyns A and D, altering the nicotinic and gamma-aminobutyric acid receptor functions, acting mainly by ingestion. Nevertheless, in an integrated pest management context, the need for the combination of several methods in order to reduce the amount of product applied and to prevent the onset of phenomena such as pesticide resistance and toxicity for non-target organisms is now clear. In this particular case, an interesting approach may rely on the integrated use of non-insecticidal control methods for the target pest to reduce selection pressures on a resistant population. Particle film technology (PFT) is a relatively new method for controlling arthropod and disease pests of food crops that is allowed in organic farming. When crops are dusted or sprayed with hydrophobic particles, a protective barrier against both plant pathogens and plant-feeding arthropods is created. In this regard, kaolin is a white, nonabrasive, fine-grained and inert aluminosilicate mineral that is purified and sized so that it easily disperses in water and creates a mineral barrier on plants that prevents oviposition and insect feeding and severely reduces insect movements by the attachment of particles to their bodies [20,21]. Previous studies have shown that hydrophobic formulations of kaolin-based particle films can effectively protect olive trees from *B. oleae*, *Prays oleae* (Bernard) and *Saissetia oleae* (Olivier) [22,23]. With particular reference to *B. oleae*, kaolin proved to have the same protective efficacy as dimethoate in a Tunisian [24] and a Portuguese [18] study, and to provide a higher degree of protection than copper at high infestation levels [25], whereas copper-based compounds are allowed to be used, with some limitations-, in organic farming and commonly used in integrated and conventional pest management. Despite its deterrent (insects move away after contact) action, kaolin is not included among the antiparasitic substances, but was admitted as a biostimulant in organic legislation.

In light of the above, the main objective of this study was to evaluate the effect, either alone or in combination, of kaolin and spinosad against *B. oleae* and its main naturally occurring parasitoids in an experimental organic olive grove.

## 2. Materials and Methods

### 2.1. Experimental Design

The study was carried out during the 2019–2020 period in a CREA OFA/ARSAC (Regional Company for the development of Calabrian agriculture) experimental field located in Mirto (39.615974, 16.7771879), Calabria Region, Italy, managed according to the provisions contained in EU Regulation 848/2018 on the production of organic food and feed. The 0.75 ha olive grove was made up of plants of the cultivar Carolea at 4 × 4 m spacing. Carolea is one of the most important and widespread Calabrian varieties, whereas Calabria is the second major olive producing region in Italy [26]. Carolea is a highly susceptible cultivar to the olive fruit fly e.g., [27,28], and thus the monocultivar condition in a single environment allowed us to limit the influence of external factors affecting *B. oleae* infestation. This aspect is particularly important considering the vast Italian olive germplasm including over 800 accessions [29], with different degrees of susceptibility to olive fly, e.g., [27,30,31].

**Figure 1:**
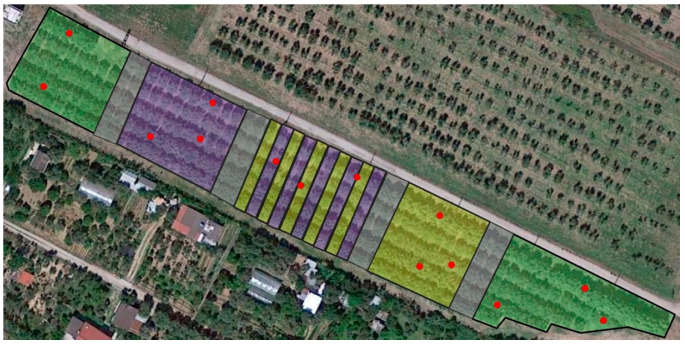
Subdivision of the experimental field according to the treatment. Highlighted in green: control; purple: kaolin; yellow: spinosad; grey: buffer zones. The red spots indicate the positioning of the chromotropic adhesive traps.

The orchard was split into five parcels consisting of two control plots interspersed by three blocks, differently treated with kaolin (K), spinosad (S) and with kaolin and spinosad (K + S) in alternate rows, respectively, as shown in Figure 1. An untreated buffer zone of two rows of olive plants divided each pair of contiguous parcels. A spinosad-based protein bait with specific attractive substances (commercial product: Spintor™ Fly; Dow Agro-Sciences LLC, Indianapolis, IN, USA) was administered at a dose of 1 L of product/ha and diluted in 4 L of water, every 6–10 days from 8 August to 11 November 2019 and from 27 July to 23 October of 2020. It was sprayed on all the perimeter plants and to alternate plants in the internal rows, in a canopy portion free from drupes and facing south.

Kaolin was administered at a dose of 5 kg/ha with three treatments (early August, early September and early October in 2020 and end of July, mid-August, and mid-October in 2020) defined on the basis of the rainy events that occurred in the two years, and in accordance with [18,32].

Temperature data were recorded by a meteorological station situated a few hundred meters away from the experimental olive orchard but within the ARSAC property.

Active (the presence of eggs and the first and second instar larvae), harmful (third instar larvae, pupae and abandoned tunnels) and total infestation (all the alive or dead preimaginal stages, parasitized and abandoned tunnels) were determined by examination samples of 100 olive drupes per treatment (C, K, S and K + S) and per sampling date under a binocular microscope, randomly collected every 7 to 10 days from August to November of each year. The effectiveness of the two products and their combined action was assessed in terms of active, harmful, and total infestation in the treated blocks compared to the control parcels.

The presence and population trend of *B. oleae* adults were monitored during the study period every 8–10 days through the placement of 14 chromotropic adhesive traps throughout the five plots so as to have a global coverage of the orchard (Figure 1).The traps consisted of yellow plexiglass panels measuring 15 × 20 cm wrapped with a transparent sticky film that was replaced at each monitoring (8–10 days). They were positioned in the medium-low part of the canopy and hooked to a branch on the most exposed side of the tree to make them more visible to the insects.

Male and female captures were discriminated by treatment in order to evaluate them for a possible repulsive effect.

### 2.2. Identification and Population Dynamic Monitoring of Naturally Occurring B. oleae Egg/Larval Parasitoids

The presence of the main natural antagonists of the olive fly during the experimental trial was monitored through different approaches: (i) adult captures through the chromotropic adhesive traps positioned for monitoring olive fly population dynamics; (ii) evaluation of the level of ectoparasitism in actively infested drupes; and (iii) evaluation of the level of endoparasitism through the collection of *B. oleae* puparia from infested drupes maintained in Petri dishes at laboratory conditions (24 ± 1 °C, 60 ± 10% RH) until the eventual parasitoids emergence from olive fly puparia. Captured/reared adult parasitoids were stored at −20 °C and then observed under the stereomicroscope for taxonomic identification by examining their morphological characteristics.

The percentage of ecto- and endoparasitism was calculated as follows:

% ectoparasitism = (number of parasitoids feeding on *B. oleae* eggs-larvae found in infested drupes/number of *B. oleae*-infested drupes) × 100 (1)

% endoparasitism = (number of parasitoids emerging from *B. oleae* puparia/number of *B. oleae* puparia) × 100 (2)

### 2.3. Statistical Analysis

The effects of treatments on the total male and female captures and fruit damages in the two years were initially tested through a factorial ANOVA with interaction effects performing the “aov” function in the “stats” package. Since only the factor “treatment” was significant, we proceeded to the analysis with a one-way ANOVA with the same package and function. Normality and homoscedasticity of residuals were tested using a Shapiro–Wilk test (function “Shapiro.test” in package “dplyr”) and Bartlett’s test (function “bartlett.test” in package “stats”). After residue analysis, data were log(x + 1) transformed in order to avoid heterogeneity of variances. Statistical significance was considered at *p* ≤ 0.01 and *p* ≤ 0.05.

A post hoc multiple comparison of means at confidence levels of 99 and 95% was made using a Tukey’s test (function “TukeyHSD” in package “stats”). All analyses were done using R 3.4.1 software [33].

## 3. Results and Discussion

### 3.1. B. oleae Population Dynamics

**Figure 2:**
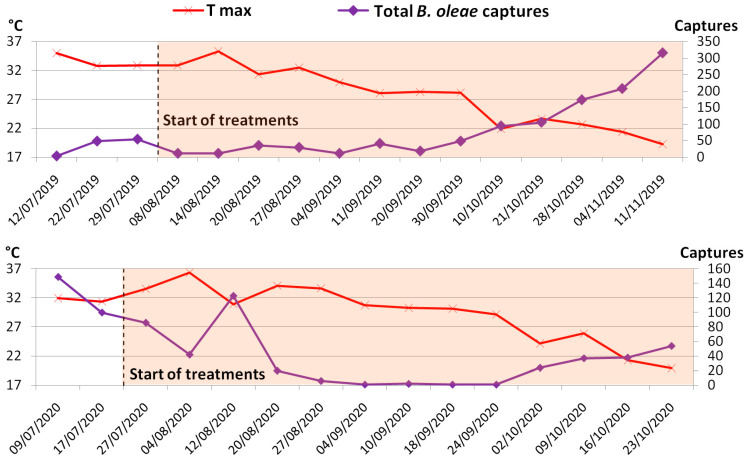
Trend of maximum temperatures and total captures of individuals of *B. oleae* in the 14 sticky traps scattered in the olive grove recorded during the two-year trial.

The overall (males plus females) trend of *B. oleae* presence in the organic olive orchard during the July to November and July to end of October periods in 2019 and 2020, respectively, is presented in Figure 2. Temperature, and in particular maximum temperature, seemed to represent the principal factor driving the increase and decrease of individuals in 2019, with maximum temperatures above 35 °C causing a drastic decline in the total number of olive fruit flies. A 35 °C threshold as a lethal temperature has been previously highlighted in a laboratory-scale study [34], even if in open-field conditions other variables, primarily air relative humidity, intervene in determining the survival/mortality rate. Accordingly, a negative correlation (Pearson’s r = −0.84) was found between maximum temperatures and *B. oleae* catches. A similar observation was also valid for 2020 until August, whereupon the drop in temperatures did not support an increase in the population of *B. oleae* for the whole month of September (Figure 2). Since this anomalous trend was uniformly found in the five parcels and considering that the orchard is located in a highly olive-vocated area, we are led to hypothesize that the reason for the zeroing of the number of catches, accompanied by an equally low rate of active infestation, might be sought in the use of insecticides in response to the peak recorded on 12 August 2020 in the adjacent orchards, having had an impact on the total population of the olive fly.

The higher number of catches found at the beginning of July 2020 compared to the same period of 2019 was likely due to the rains that occurred during the month of June 2020 and which are the basis of the different population trends observed in the two years. In this sense, the highest values were recorded in November in 2019 with 524 catches and in July in 2020 with 335 catches. The resulting highest values per single trap were recorded on 11 November 2019 (56) and on 9 July 2020 (61).

**Figure 3:**
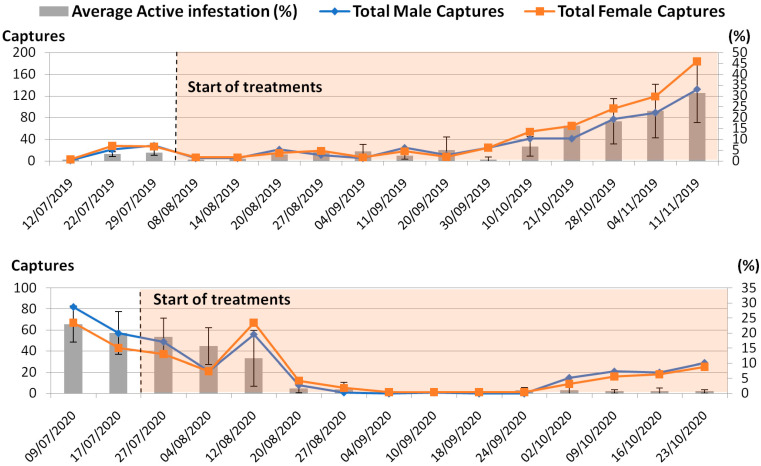
Trend of average active infestation (with standard deviation bars) and total captures of male and female individuals of *B. oleae* during the two-year trial, regardless of treatment.

Regarding sex ratio, a balanced distribution was observed via adult captures through chromotropic adhesive traps (Figure 3) in both years, while, as easily predictable, a strong correlation (Pearson’s r = 0.96 and 0.80 for 2019 and 2020, respectively) was found between female captures and active infestation, namely representing the most “recent” portion of the infestation, including the presence of eggs and the first and second instar larvae inside the drupes’ pulp. Regarding the positioning of the traps, in the untreated period, a statistically significant difference (*p* = 0.046) in *B. oleae* captures was only found between the parcels subsequently sprayed with kaolin and spinosad (with higher values in the former) in the two years, with a consequent general uniform spread of this dipterous.

### 3.2. Effect of Treatment on Drupes’ Infestation Level and B. oleae Captures

**Figure 4:**
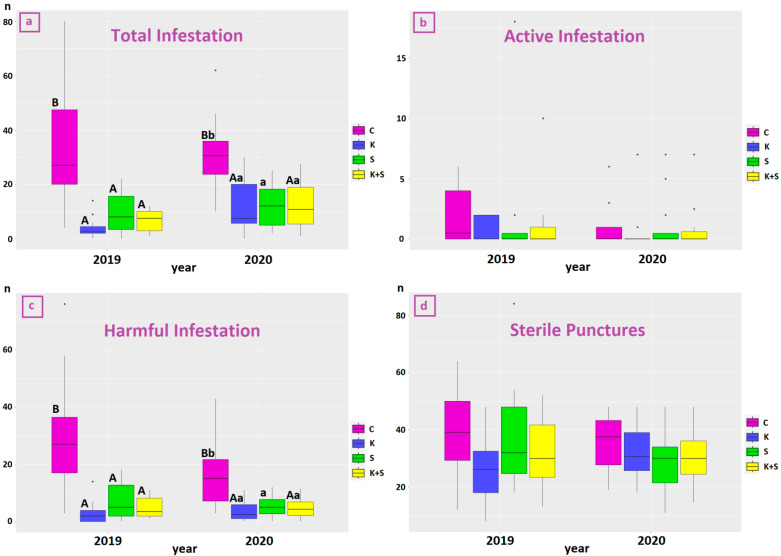
Box plots showing the level of (**a**) total, (**b**) active and (**c**) harmful infestation and (**d**) the number of sterile punctures over the two year (2019 and 2020) experiment. Control is shown in purple, kaolin in blue, spinosad in green, and synergic in yellow. Different uppercase letters indicate statistical significance at 99% level; different lowercase letters indicate statistical significance at 95% through post-hoc multiple means comparison by Tukey’s honestly significant difference (HSD) test. Years have been analyzed separately.

The total effect of treatments on total, active and harmful infestation over the two years (2019 and 2020) is shown in Figure 4.

The comparison of the two years shows that 2019 had higher levels of total infestation than 2020 (*p*-value = 0.006) regardless of treatment, but no significant differences were found among single treatments by considering interaction effects. Total infestation was statistically significantly higher in the control parcels in both years compared to the treated ones. No difference emerged from the comparison of the effect given by the three treatments with each other (Figure 4a). Active infestation was not overall significantly affected by treatment either in 2019 (*p*-value = 0.691) and in 2020 (*p*-value = 0.869), with tendentially higher values in the untreated plot (C, Figure 4b), while a Tukey’s test showed that either in 2019 and in 2020 the three treatments (K, S and K + S) were significantly different from the control for harmful infestation, presenting lower values with a comparable protective effect in a highly susceptible cultivar such as Carolea (Figure 4c). Eventually, the treatment did not seem to influence the number of sterile punctures.

Regarding treatment, in general kaolin showed the best results, with lower infestation levels in absolute value; which was even better considering that the highest numbers of fly captures before treatments were recorded in this parcel. This finding is in accordance with the work by Rizzo and collaborators [35] recording a higher efficacy of kaolin against *B. oleae* compared to a spinosad-based bait in three Sicilian organic olive orchards planted with the medium-high susceptible cvs Cerasuola and Nocellara del Belice, without however testing their combined use. Similarly, field experiments on the cv Carolea demonstrated a greater effectiveness of kaolin compared to rotenone [36], copper and copper mixed to kaolin [37]. These studies also revealed the neutral effect of kaolin on the chemical and sensory characteristics of the extracted oils. Conversely, kaolin (as well as copper) treatment was demonstrated to affect the sensorial attributes of table olives and the microbial population during fermentation [38].

Nevertheless, in the present study, K, S and K + S have proven to be good alternatives (and also interchangeable), with similarly effective results, especially considering that we are led to believe that the actual effectiveness of the treatments is somehow masked by the limited presence of the olive fly detected in several lapses of time in the biennium of the study. This evidence is particularly important for olive growers in the definition of olive fruit fly management systems aimed at avoiding the generation of selection pressures, at limiting prolonged effects on (even beneficial) non-target organisms and, no less important, at cost reduction through a rationalization in the use and dosage of control methods. In fact, in orchard experiments, the onset of resistance to spinosad in olive fly [39], as well as toxicity for non-target insects [40,41,42] has been described, whereas the drawbacks related to kaolin employment concern the onset of resistance [38], leaching by rainfall (and so the need for repeated applications during the olive season), and the negative effects on abundance and the diversity of non-target arthropods [23,43,44] and the community of natural enemies [25]. However, this negative effect could be somewhat mitigated through the sustainable management of the olive grove (such as organic management) that has clear positive effects on some soil quality parameters including microbial and pedofaunal communities abundance and diversity [45,46,47].

As for the male and female catches, no significant differences between treatments were highlighted (*p*-value > 0.05) in either year, indicating no particular deterrent effect on *B. oleae* flight of either kaolin or spinosad. In the overlapping period of experimentation of the two years (August 8–October 23), the average total catches for single traps did not show major differences, ranging from 31.66 ± 36.12 (C) to 15.333 ± 15.41 (K) to 30.16± 27.12 (S) to 22.75 ± 15.87 (K + S) in 2019, and from 15 ± 4.6 (C) to 18.83 ± 21.03 (K) to 25.33 ± 27.50 (S) to 22.08 ± 7.66 (K + S) in 2020.

### 3.3. Identification and Monitoring of Naturally Occurring Parasitoids of B. oleae

**Figure 5:**
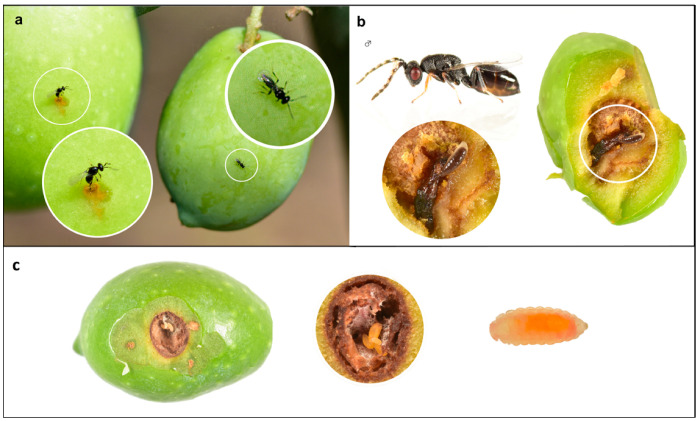
Images of the three different naturally occurring ectoparasitoids found in the experimental organic olive grove. (**a**): Female adults of *Eupelmus urozonus* in the act of egg-laying in a oviposition puncture of *B. oleae.* (**b**): Larva of *Eurytoma martellii* feeding on a third instar larva of *B. oleae* and a captured female adult. (**c**): Larvae of *Prolasioptera berlesiana* feeding on the mycelium of the symbiotic fungus *Botryosphaeria dothidea* within the olive fruit mesocarp.

**Figure 6:**
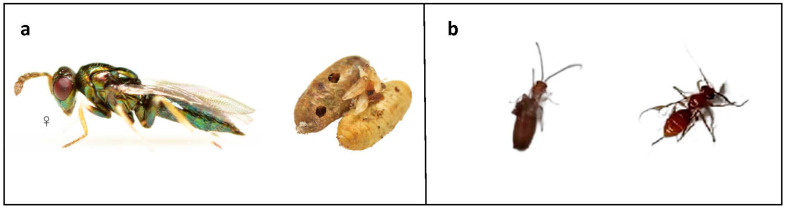
Images of the two different naturally occurring endoparasitoids found in *B. oleae* puparia from infested drupes of Carolea cv. (**a**): Pupae of *Baryscapus sylvestrii* emerged from pupae of *B. oleae*. (**b**): Adult of *Psyttalia concolor* emerging/emerged from *B. oleae* puparium.

The morphological examination of captured and/or reared adults allowed for the verification of the presence of five natural antagonists of the olive fly. Specifically, three ectoparasitoids (Figure 5): *Eurytoma martellii* Domenichini (Hymenoptera: Chalcidoidea, Eurytomidae)*, Prolasioptera berlesiana* Paoli (Diptera: Cecidomyiidae) and *Eupelmus urozonus* Dalman (Hymenoptera: Chalcidoidea, Eupelmidae), and two endoparasitoids (Figure 6): *Psyttalia concolor* Szépligeti (Hymenoptera: Braconidae)*,* and *Baryscapus sylvestrii* Viggiani& Bernardo (Hymenoptera: Chalcidoidea, Eulophidae), were identified. As regards the latter, it was during this trial that this insect was first observed in Calabria [48].

The number of adult captures and the level of endo- and ectoparasitism in infested drupes turned out to be quite homogeneous in the differently managed parcels (Table 1) without statistically significant differences among the treatments. Nonetheless, as this was largely predictable, indigenous parasitoids did not seem to significantly impact the population size of *B. oleae*. However, the mass introduction of natural antagonists could be part of an integrated control strategy, also allowed under the organic legislation, although it needs to be said that a significant limitation in this regard is represented by a generally inefficient parasitoids’ mass rearing [49]. The higher number of insects emerging from puparia collected in the control plot only depends on the higher number of puparia found in the drupes of this parcel. This study suggests a harmless effect of kaolin and spinosad against the indigenous *B. oleae* antagonists. Therefore, kaolin’s deterrent effect is likely only limited once the fly has laid its eggs inside the drupes. These results confirm the neutral effect of kaolin on *P. concolor,* as demonstrated in controlled environments [50,51], as well as in the open field [24]. Laboratory tests highlighted the non-harmful nature of kaolin against the *B. oleae* parasitoids *P. concolor* and *Chrysoperla carnea,* albeit with some effect on their fecundity, and other beneficial insects of the olive grove agroecosystem [52], as well as against the natural enemies’ communities [53].

Regarding spinosad, some evidence of moderately harmful or harmful effects on parasitoids have been reported [54,55,56]; however, for *B. oleae* antagonists, these have been described in laboratory experiments but not in field ones [57]. Notwithstanding this, it cannot be excluded that the lower values on the number of parasitoids found in the spinosad-treated plot could be attributed to its residual toxicity, which lasted from three to ten days after treatment [50].

Lastly, a separate discussion is required for *Prolasioptera berlesiana* female that, with oviposition, also inoculates its symbiotic fungus *Botryosphaeria dothidea* (anamorph of *Fusicoccum aesculi*) inside the drupe via *B. oleae* puncture, causing necrotic spots and depressions on the fruit [58]. This primarily visual damage known as fruit rot can cause significant production losses, especially for table olives, but can simultaneously favor the onset of other diseases [59]. Although *P. berlesiana* is not the exclusive source of inoculum of the fungus [60] (and even the fungi *Neofusicoccum australe* and *N. vitifusiforme* have been associated to olive fruit rot symptoms [61]), a more in-depth analysis of the actual economic and ecological benefits must be carried out before the employment of this dipterous in the biological control of *B. oleae.* In this study, symptoms of fruit rot were evident in 3.77, 2.33, 2.41 and 2.28% of the drupes collected in C, in K, S and K + S, respectively, implying significant losses for a double aptitude (for oil and table olives) cultivar such as Carolea.

## 4. Conclusions

*Bactrocera oleae* is responsible for remarkable quantitative (and qualitative) yearly losses in the olive orchards worldwide. Thus, the adoption of low environmental impact containment strategies is a key point in the organic sector, made even more difficult by the limited choice of products permitted by the current legislation. In this sense, in this two year trial, kaolin and spinosad have been found to be effective in limiting total and harmful fly infestation, even in combination, thus reducing application costs and preventing phenomena such as the onset of resistance and prolonged harmful exposures for non-target organisms. Moreover, these treatments did not appear to have adverse effects on the indigenous natural antagonists of *B. oleae,* thus paving the way for the investigation of case-specific integrated pest management strategies. This information is even more interesting considering the need for alternatives to replace the withdrawn traditionally used pesticides in conventional oliviculture. Further experimental tests are needed, as the fight against *Bactrocera oleae* is a continuing one.

## Figures and Tables

**Table 1:** Number of total (captured + reared) individuals collected and percentage of endo- and ectoparasitism for the five naturally occurring *B. oleae* parasitoids found in the olive orchard.

Parasitoid	Treatment	Total Individuals		% of Endoparasitism		% of Ectoparasitism	
		2019	2020	2019	2020	2019	2020
*Eurytoma* *martellii*	C	5	7	2.02	2.23		
	K	1	4	2.08	3.17		
	S	1	3	1.67	2.46		
	K + S	2	4	1.85	2.31		
*Prolasioptera* *berlesiana*	C	9	11	3.03	3.36		
	K	4	5	4.17	3.17		
	S	3	6	3.33	3.28		
	K + S	3	6	3.70	3.84		
*Eupelmus* *urozonus*	C	4	6	1.51	1.86		
	K	1	3	2.08	2.38		
	S	1	4	1.67	2.46		
	K + S	1	3	1.85	1.54		
*Psyttalia* *concolor*	C	12	6			5.78	3.48
	K	3	1			6.89	3.84
	S	1	1			2.78	0
	K + S	2	1			2.63	3.12
*Baryscapus* *sylvestrii*	C	8	6			4.04	4.65
	K	1	3			3.44	3.84
	S	2	2			2.77	2.5
	K + S	2	1			5.26	3.12

## Data Availability

Data sharing not applicable.
